# Perinatal Outcome and Its Association with Blood Pressure Levels in Women with Preeclampsia

**DOI:** 10.3390/jcm11216334

**Published:** 2022-10-27

**Authors:** Daniela Willy, Ralf Schmitz, Walter Klockenbusch, Helen Ann Köster, Kevin Willy, Janina Braun, Mareike Möllers, Kathrin Oelmeier

**Affiliations:** 1 Department of Obstetrics and Gynecology, University Hospital Münster, 48149 Münster, Germany; 2 Department of Cardiology, University Hospital Münster, 48149 Münster, Germany

**Keywords:** preeclampsia, arterial hypertension, delivery mode, induction of labour, preterm delivery

## Abstract

Background: Timing and mode of delivery in women with preeclampsia remains challenging, often balancing the risk of severe maternal complications and preterm delivery with its risks for the newborn. It is known that women with very high blood pressure levels in pregnancy have more unfavourable outcomes, but there is little data on neonatal outcome in these cases and the effect of the delivery mode. Methods: We included 158 preeclamptic women in our single-centre retrospective cohort study. Patients were divided into three subgroups depending on blood pressure levels, and delivery mode as well as neonatal outcomes were analysed. Furthermore, the effect of gestational age at delivery was assessed. Results: Maternal blood pressure levels correlated negatively with gestational age at delivery (*p* = 0.007) and positively with delivery via caesarean section (*p* = 0.003). Induction of labour was more frequent in women with lower blood pressure levels (*p* = 0.008) and higher gestational age (*p* < 0.001). If labour was induced, vaginal delivery was achieved equally often in all gestational ages. Neonatal outcome appears to be more favourable after vaginal delivery compared to planned caesarean section (*p* < 0.001). Conclusions: Induction of labour should be discussed generously in preeclamptic women, even if blood pressure levels are high and/or gestational age is young, as success rates seem to be adequate and neonatal outcome is more favourable after vaginal delivery. Large prospective trials are needed to better evaluate success rates, risks and complications of induced labour and the effects of delivery mode on neonatal outcome in preeclampsia.

## 1. Background

Preeclampsia remains one of the most dangerous complications of pregnancy for mother and child. It is defined as arterial hypertension in or after the 20th week of gestation in combination with significant proteinuria and/or onset of other organ dysfunction [1,2].

Therapeutic options remain limited with delivery being the only causal therapy. Consequently, preeclampsia is a common cause for preterm delivery to avoid severe complications for mother and child [1]. Unfortunately, preterm delivery is associated with many risks and potential long-term consequences for the newborn, e.g., cerebral palsy, chronic lung disease, necrotizing enterocolitis, retinopathy of prematurity. The earlier the gestational age at delivery, the higher the risk of these complications occurring [3]. Timing of delivery in women with preeclampsia remains challenging, balancing the risk of maternal against neonatal complications [4,5]. When delivery is indicated, the best mode of delivery must be discussed. In uncomplicated singleton pregnancies, caesarean sections are known to cause more maternal complications compared to vaginal deliveries [6]. It is unclear whether this is also valid for women with preeclampsia, especially in cases of preterm delivery. Moreover, data on neonatal outcome in this special situation remain sparse and the correct management is still uncertain [7].

Preeclampsia can be classified into preeclampsia with and without severe features, as well as early- and late-onset preeclampsia. Severe features of preeclampsia include systolic blood pressure ≥ 160 mmHg, diastolic blood pressure ≥ 110 mmHg, thrombocytopenia, impaired liver function, renal insufficiency, pulmonary oedema, neurological or visual disturbances [8]. Early-onset preeclampsia is defined as preeclampsia starting before 34 weeks of gestation, whereas late-onset preeclampsia develops at or after 34 weeks of gestation [9].

No uniform recommendations exist for timing and mode of delivery in women with severe preeclampsia, especially when it comes to (very) preterm delivery.

Current international guidelines on arterial hypertension grade hypertensive blood pressure levels into different degrees of severity, partly because patients with higher blood pressure levels are at higher risk for complications [10]. Moreover, in pregnant women, Buchbinder and colleagues showed that perinatal outcomes are more favourable in mild preeclampsia compared to severe hypertension [11]. This led us to the question whether a classification of preeclampsia into different degrees of severity according to maternal blood pressure levels could be helpful for perinatal risk stratification. In a previous study, it could be shown that the level of hypertension in preeclamptic women is an independent marker for maternal outcome [12]. It remains uncertain if there is a direct impact on the mode of delivery and neonatal outcome. We therefore assessed the mode of delivery, subsequent complications and neonatal outcome in relation to maternal blood pressure levels and gestational age. We suspected that women with higher blood pressure levels and early-onset preeclampsia would undergo planned caesarean sections more often, possibly leading to more unfavourable outcomes.

## 2. Methods

A retrospective cohort study at the University Hospital Münster, a tertiary obstetric centre, was conducted from 1 January 2017 to 31 December 2020. The study was designed according to the Declaration of Helsinki and approved by the institutional review board. All women giving birth within this period were included in the analysis if preeclampsia was diagnosed. The definition of preeclampsia was implemented as described by the International Society for the Study of Hypertension in Pregnancy (ISSHP) and in the current ACOG practice bulletin and AWMF-guideline [13,14,15].

During the hospital stay demographic data, medical history, blood pressure levels, laboratory results and maternal as well as neonatal outcomes were recorded.

In conformity with current international guidelines for arterial hypertension, we divided the patients into three subgroups depending on their maximum blood pressure [10,16]:

- moderate hypertension: systolic blood pressure < 160 mmHg and diastolic blood pressure < 109 mmHg
- severe hypertension: systolic blood pressure 160–179 mmHg and/or diastolic blood pressure 110–119 mmHg
- hypertensive crisis: systolic blood pressure ≥ 180 mmHg and/or diastolic blood pressure ≥ 120 mmHg

Moreover, we analysed outcomes depending on delivery mode and relation to gestational age in our patient cohort, and therefore made use of the common classification for preterm birth, depending on the gestational age at delivery [17,18]:

- extremely preterm: < 28 weeks of gestation at delivery
- very preterm: 28 to 32 weeks of gestation at delivery
- mild or moderate/late preterm: 32 to 37 weeks of gestation at delivery.

Foetal growth restriction (FGR) was defined as estimated birth weight below the 10th percentile and/or foetal growth below the estimated percentile curve and pathological uterine artery or umbilical artery Doppler or oligohydramnios [19].

An induction of labour was offered to patients who were physically capable of and agreed with an induction of labour and if no obstetrical contraindications for a vaginal delivery were present. For induction of labour, we used dinoprostone gel, administrated misoprostol orally or oxytocin intravenously. The dosage chosen was determined by the contraction pattern and in agreement with the patient’s wishes. Medication for induction of labour, mode of administration and dosages are demonstrated in Supplementary Table S1.

## 3. Statistical Analysis

All statistical calculations were performed using SPSS Statistics, version 27 (IBM, Armonk, NY, USA). For descriptive data analysis, we provided mean values and standard deviation for continuous variables. Categorical data are expressed as frequencies/percentages. For comparison of two ordinally scaled variables, we used chi-squared test after constructing contingency tables. We used chi-squared test to express odd’s ratio. After proving variance homogeneity with Levene’s test, we tested for correlation between groups with a one-way ANOVA. Student’s *t*-test was used to evaluate the differences in mean of metric variables between groups. When performing multiple comparisons, Bonferroni correction was applied.

A *p*-value < 0.05 was considered statistically significant, and significance levels were presented as follows: *p*-values < 0.05 are summarized with one asterisk (*).

## 4. Results

From 1 January 2017 to 31 December 2020, 5149 women gave birth at the University Hospital Münster. Of these, 158 were diagnosed with preeclampsia (3%) and were included in this study.

These 158 included women had a mean age of 31.9 ± 5.1 years. The majority of the women were primiparous (69.0%). Most patients were from Germany, while 18.3% were from other countries. Pre-existing arterial hypertension was known in 15.8% of all patients included, 11.4% had a coagulation disorder and 3.8% a history of thrombosis, respectively. Pre-existing diabetes mellitus was documented in 5.1% of the patients in this cohort, whereas 19.6% were diagnosed with gestational diabetes during this pregnancy. A fertility treatment was performed in 11.4% of all cases.

More than one quarter of all foetuses had an estimated birth weight under the 10th percentile (27.8%), most of these (21.5% of total) met the criteria of FGR.

Of the 158 included pregnancies, 22 (13.9%) were twin pregnancies. No significant correlation could be found between multiple pregnancies and the following: maternal blood pressure, gestational age at delivery, induction of labour and mode of delivery. We therefore did not differentiate between single and multiple pregnancies in the further analysis.

We then divided patients into different subgroups, depending on their blood pressure levels. Subgroup-specific patient characteristics are displayed in Table 1.

We found that maternal blood pressure levels correlated negatively to the gestational age at delivery (*p* = 0.007). Preeclamptic women with hypertensive crises were more likely to deliver a preterm baby than women with only moderate hypertension (*p* = 0.001). When regarding the different blood pressure groups, women with moderate hypertension delivered a mean 19 days later than women with hypertensive crises (Table 2). The birth weight of neonates whose mothers had hypertensive crises during pregnancy was significantly lower than those of women with only moderate hypertension. When looking at FGR, we found a tendency for higher numbers of FGR in women with hypertensive crises, but it did not reach statistical significance (Table 2). There were no significant differences in birth weight percentiles. 

When analysing the different blood pressure groups and correlating them with the stage of preterm/term delivery, we also found significant associations, as shown in Table 3.

**Figure 1:**
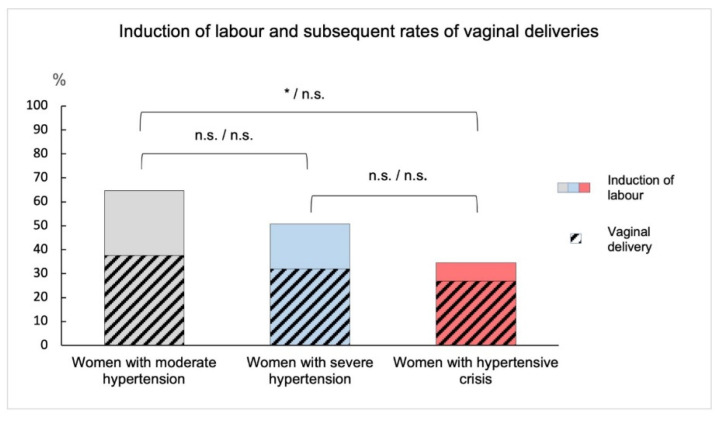
Induction of labour and subsequent rates of vaginal deliveries in women with preeclampsia. The rates of induction within the three blood pressure groups and resulting proportion of vaginal deliveries are shown. Induction of labour was significantly more frequent in women with moderate hypertension compared to women with hypertensive crises (* = *p* = 0.03), all other comparisons are not significant (n.s.).

We analysed the mode of delivery and whether labour was induced and correlated this to the maternal blood pressure levels and gestational age. Induction of labour was significantly more frequent in women with moderate hypertension compared to women with hypertensive crises (*p* = 0.03; see Figure 1). The likelihood of vaginal delivery after medical induction of labour was similar in all three groups with no significant differences: Of the 31 women induced with moderate hypertension, 18 delivered vaginally (58.1%); of 35 women with severe hypertension, 22 delivered vaginally (62.9%); and of 15 women induced in the hypertensive crisis group, 11 women had a vaginal delivery (73.3%).

We found no significant differences between rates of induction of labour in primiparous vs. multiparous women, but we observed a higher rate of caesarean sections in primiparous compared to multiparous women (*p* = 0.03).

We found a significant correlation between maternal blood pressure levels and the mode of delivery: the higher the maternal blood pressure, the more likely a delivery via planned caesarean section was (*p* = 0.003). Conversely, the rate of spontaneous vaginal delivery was similar in all three blood pressure groups (no significant differences). Operative vaginal delivery was significantly more frequent in women with moderate or severe hypertension than in women with hypertensive crises. Delivery via unplanned caesarean section (secondary caesarean section) was most common in women with moderate hypertension (Table 4).

**Figure 2:**
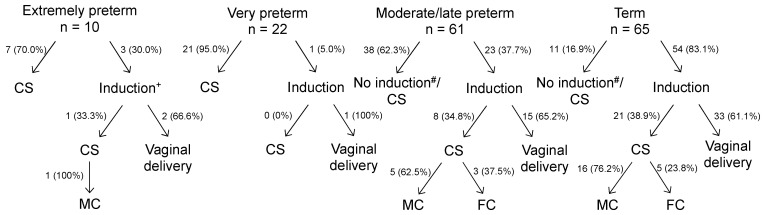
Rates of labour induction and consequent vaginal delivery compared to delivery via caesarean section (CS) in women with preeclampsia, division according to gestational age at delivery. Furthermore, reasons for performing CS after induction are classified as caused by maternal condition (MC) or foetal condition (FC). ^+^ = foetus non-viable ^#^ = one patient in each group had a spontaneous vaginal delivery, all other patients had a CS.

An induction of labour was more frequent in women with higher gestational age compared to women with (very) preterm infants (*p* < 0.001), regardless of maternal blood pressure classification. The rate of labour induction and subsequent vaginal delivery within the gestational age groups is shown in Figure 2. Altogether, an induction of labour was less frequently attempted in pregnancies of younger gestational age, but if it was attempted, vaginal delivery was achieved equally often.

We found no differences regarding the rate of emergency caesarean sections between the three groups.

Gestational age at delivery did not correlate with umbilical cord pH levels, but it did correlate with APGAR values: the earlier the gestational age at delivery, the lower the average APGAR values at 5 and 10 min (*p* < 0.001). Low gestational age at delivery was also positively associated with an admission to the neonatal care unit after birth (*p* < 0.001).

No correlation could be shown between maternal blood pressure levels and APGAR values or pH levels in the umbilical cord; nor did maternal systolic blood pressure levels correlate with foetal growth restriction. However, we did find that the children of women with high diastolic blood pressure levels suffered from growth-restriction significantly more often (*p* = 0.01). The higher the maternal blood pressure levels, the more often the newborn was admitted to the neonatal care unit after birth (*p* = 0.04).

The pH levels in the umbilical cord were not influenced by the mode of delivery, but we did find a significant correlation between the mode of delivery and APGAR values: APGAR values after 5 and 10 min were significantly higher after vaginal delivery than after planned caesarean section (*p* = 0.001). Moreover, children were admitted to the neonatal care unit more frequently after a planned caesarean section than after a vaginal delivery (*p* = 0.001).

## 5. Discussion

Succinctly, we found that high maternal blood pressure levels in preeclamptic women correlate with preterm delivery. Women with hypertensive crises are at highest risk for extremely preterm delivery. An induction of labour was performed less often in women with hypertensive crisis compared to those with moderate hypertension, but if it was performed, vaginal delivery was achieved equally often. On the other hand, we could show that rates for planned caesarean section were highest in women with hypertensive crises, even though rates for spontaneous vaginal delivery were similar in all groups. Induction of labour was performed more frequently in women with higher gestational age than in women with preterm infants. However, if an induction of labour was performed, vaginal delivery was achieved equally often in all groups. Neonatal outcome appears to be more favourable after vaginal delivery compared to planned caesarean section, but it should be taken into account that the average gestational age of children delivered vaginally was higher than those delivered via caesarean section.

Several of these findings are consistent with results from other studies: Proussaloglou et al. concluded that women with severe hypertension have higher associated maternal and neonatal adverse events [20], and O’brien and colleagues showed that most women with high blood pressure levels delivered via caesarean section [21]. 

Other studies showed that a hypertensive disorder of pregnancy was a predictor for an unsuccessful induction of labour, resulting in higher rates of caesarean sections, independent of gestational age and parity, while nonetheless most women with preeclampsia deliver vaginally [22,23]. In contrast to this, Durst et al. outline that an early induction of labour did not increase rate of caesarean sections in women with a hypertensive disorder in pregnancy [24]. We did not find any data analysing whether the severity of hypertension in pregnancy influences timing and mode of delivery, or the frequency and success of induced labour. Our results show that in women with preeclampsia, blood pressure levels are related to the gestational age at delivery, while the rate of spontaneous vaginal deliveries was not affected by maternal blood pressure levels.

When regarding the mode of delivery and the gestational age in preeclampsia, there is interesting pre-existing data. Thornton et al. came to the conclusion that women with preeclampsia <33 weeks of gestation had lower perinatal mortality rates when a planned caesarean section was performed, whereas for women ≥33 weeks of gestation lower perinatal mortality rates were observed when a vaginal delivery was induced [25]. In contrast to that, Alanis and colleagues found that neonatal outcomes were not worsened by an induction of labour in severe, early-onset preeclampsia, but stated that if it was attempted, it was often unsuccessful when gestational age was <28 weeks [26]. A study of Coviello et al. also showed that rates of vaginal delivery after induction increase with gestational age. Moreover, this group found no differences in maternal and neonatal adverse events between vaginal delivery and planned caesarean section [27]. Van Eerden and colleagues reviewed the delivery mode in severe preeclampsia before 28 weeks of gestation and concluded that maternal and neonatal outcomes were not affected by the delivery mode. They also suggested discussing labour induction for women with severe preeclampsia before 28 weeks of gestation but emphasized small case numbers for this special situation [28]. Another study did not show a favourable outcome for women with severe preeclampsia and immediate delivery via caesarean section, in fact it showed more pulmonary complications in mothers and newborn after caesarean section than after vaginal delivery [29]. Taken together, no consensus exists about a clear threshold regarding gestational age for induction of labour or performing primary caesarean section in women with early-onset preeclampsia. The literature and our analysis rather support an individualized management in which health status of mother and child as well as severity of preeclampsia should guide the decision-making process [26,28]. This process should involve obstetricians as well as neonatologists and, if necessary, further disciplines (anaesthesiology, internal medicine). When regarding women at or near term with preeclampsia, induction of labour seems to be associated with an improved maternal outcome and does not increase rates for unplanned caesarean sections [30,31].

The limitations of our study are the relatively small case numbers in the extremely and very preterm groups, and the retrospective design. The choice of the mode of delivery and the success rate for a vaginal delivery depend on different factors, such as history of prior deliveries. Due to relatively small patient numbers and the division into subgroups, a further statistical analysis of these factors could not reliably be performed, which is a possible weakness of this trial.

Moreover, a longer and more distinct follow-up of neonatal outcomes would have been interesting and might strengthen the findings of this study. A strength is the division into different subgroups according to maternal blood pressure levels, and distinct analysis of the mode of delivery and gestational age at delivery.

## 6. Conclusions

We conclude that an induction of labour should always be considered in women with preeclampsia, even if maternal blood pressure levels are high and/or in cases of early-onset preeclampsia with a young gestational age. Most studies mentioned above showed that adverse maternal or neonatal events are not more common if a vaginal delivery is attempted, which is in line with our findings. Only one study of Colvin and colleagues was found, which stated that a duration of labour over 24 h was associated with an increased risk for maternal and neonatal morbidity [32]. Thus, we suggest discussing induction of labour generously in women with preeclampsia if delivery is indicated, even when blood pressure levels are high and/or gestational age is young, providing there is no obstetrical contraindication for vaginal delivery. Naturally, patients must be monitored very closely, especially during prolonged induction of labour, and there will be cases in which the indication for a caesarean section will develop. Nevertheless, the consequences for future pregnancies for mother and subsequent children should be considered when initiating caesarean sections, e.g., repeat caesarean sections, placenta accreta, uterine rupture. Large prospective trials are needed to further evaluate success rates and possible effects of induced labour and the delivery mode on neonatal and maternal outcomes in women with preeclampsia, especially regarding women with high blood pressure levels and/or young gestational age.

## Figures and Tables

**Table 1:** Patient characteristics within the blood pressure subgroups.

	Group 1: Moderate Hypertension (*n* = 48)	Group 2: Severe Hypertension (*n* = 69)	Group 3: Hypertensive Crisis (*n* = 41)	Significant Pairs among Groups	Significances
mean age ± SD (years)	31.9 ± 4.5	31.1 ± 5.3	32.8 ± 5.4	none	1 vs. 2: *p* = n.s. 1 vs. 3: *p* = n.s. 2 vs. 3: *p* = n.s.
BMI ± SD (kg/m^2^)	31.1 ± 7.3	30.7 ± 7.4	32.4 ± 7.7	none	1 vs. 2: *p* = n.s. 1 vs. 3: *p* = n.s. 2 vs. 3: *p* = n.s.
nulliparous women	36 (75.0%)	49 (71.0%)	24 (58.5%)	none	1 vs. 2: OR = 1.06 *p* = n.s. 1 vs. 3: OR = 1.28 *p* = n.s. 2 vs. 3: OR = 1.21 *p* = n.s.
multiple pregnancies	9 (18.6%)	9 (13.0%)	4 (9.8%)	none	1 vs. 2: OR = 1.43 *p* = n.s. 1 vs. 3: OR = 1.90 *p* = n.s. 2 vs. 3: OR = 1.33 *p* = n.s.
pre-existing hypertension	4 (8.3%)	7 (10.1%)	14 (34.1%)	1 vs. 3, 2 vs. 3	1 vs. 2: OR = 0.82 *p* = n.s. 1 vs. 3: OR = 0.24 *p* = 0.01 2 vs. 3: OR = 0.30 *p* = 0.01

**Table 2:** Gestational age at delivery and birth weight within the blood pressure subgroups.

	Group 1: Moderate Hypertension (n = 48)	Group 2: Severe Hypertension (n = 69)	Group 3: Hypertensive Crisis (n = 41)	Significant Pairs among Groups	Significances
gestational age at delivery (weeks) ± SD (days)	36 1/7 ± 4	35 0/7 ± 4	33 3/7 ± 4	1 vs. 3, 2 vs. 3	1 vs. 2: *p* = n.s. 1 vs. 3: *p* = 0.001 2 vs. 3: *p* = 0.03
birth weight ± SD (grams)	2604 ± 1016	2279 ± 913	2015 ± 875	1 vs. 3	1 vs. 2: *p* = n.s. 1 vs. 3: *p* = 0.001 2 vs. 3: *p* = n.s.
number of FGR	7 (14.6%)	15 (21.8%)	12 (29.3%)	none	1 vs. 2: OR = 0.67 *p* = n.s. 1 vs. 3: OR = 0.50 *p* = n.s. 2 vs. 3: OR = 0.74 *p* = n.s.

**Table 3:** Correlation of maternal blood pressure levels and gestational age at delivery. We used common classifications for subdivision into blood pressure groups as well as gestational age at delivery [10,16,18,20]. The proportion of patients from the respective gestational age group in relation to the blood pressure group is expressed in percentages (e.g., 2/48 (4.2%) women blood pressure groups between different gestational ages was compared and statistical differences were analysed.

	Extremely Preterm (*n* = 10)	Very Preterm (*n* = 22)	Moderate/Late Preterm (*n* = 61)	Term (*n* = 65)
group 1: moderate hypertension (*n* = 48)	2 (4.2%)	6 (12.5%)	12 (25.0%)	28 (58.3%)
group 2: severe hypertension (*n* = 69)	2 (2.9%)	11 (15.9%)	28 (40.6%)	28 (40.6%)
group 3: hypertensive crisis (*n* = 41)	6 (14.6%)	5 (12.2%)	21 (51.2%)	9 (22.0%)
Significant pairs among groups and significances	1 vs. 3, 2 vs. 3 1 vs. 2: OR = 1.44 *p* = n.s. 1 vs. 3: OR 0.29 *p* = 0.05 2 vs. 3: OR = 0.20 *p* = 0.02	None 1 vs. 2: OR = 0.79 *p* = n.s. 1 vs. 3: OR = 1.02 *p* = n.s. 2 vs. 3: OR = 1.30 *p* = 0.29	1 vs. 2, 1 vs. 3 1 vs. 2: OR = 0.62 *p* = 0.04 1 vs. 3: OR = 0.49 *p* = 0.004 2 vs. 3: OR = 0.79 *p* = n.s.	All 1 vs. 2: OR = 1.44 *p* = 0.03 1 vs. 3: OR = 2.65 *p* = 0.001 2 vs. 3: OR = 1.85 *p* = 0.02

**Table 4:** Maternal blood pressure levels in association with the mode of delivery.

	Spontaneous Vaginal Delivery	Operative Vaginal Delivery	Primary Caesarean Section	Secondary Caesarean Section
group 1: moderate hypertension (*n* = 48)	13 (27.1%)	7 (14.6%)	12 (25.0%)	16 (33.3%)
group 2: severe hypertension (*n* = 69)	14 (20.3%)	9 (13.0%)	38 (55.1%)	8 (11.6%)
group 3: hypertensive crisis (*n* = 41)	10 (24.4%)	1 (2.4%)	25 (61.0%)	5 (12.2%)
Significant pairs among groups and significances	None 1 vs. 2: OR = 1.33 *p* = n.s. 1 vs. 3: OR = 1.11 *p* = n.s. 2 vs. 3: OR = 0.83 *p* = n.s.	1 vs. 3, 2 vs. 3 1 vs. 2: OR = 1.12 *p* = n.s. 1 vs. 3: OR = 6.08 *p* = 0.015 2 vs. 3: OR = 5.42 *p* = 0.012	1 vs. 2, 1 vs. 3 1 vs. 2: OR = 0.45 *p* = 0.005 1 vs. 3: OR = 0.41 *p* = 0.005 2 vs. 3: OR = 0.90 *p* = n.s.	1 vs. 2, 1 vs. 3 1 vs. 2: OR = 2.87 *p* = 0.003 1 vs. 3: OR = 2.73 *p* = 0.007 2 vs. 3: OR = 0.95 *p* = 0.46

## Data Availability

Raw data is available upon reasonable request from the corresponding author.

## Supplementary Materials

The following supporting information can be downloaded at: https://www.mdpi.com/article/10.3390/jcm11216334/s1, Table S1. Medication used for induction of labour. Shown is the mode of administration and the dosages used. Daily dosage was determined by the contraction pattern and the progress in labour. Figure S1. ROC Curve for association between maximum blood pressure and gestational age at delivery. AUC = 0.615 [0.518; 0.713], *p* < 0.022. Figure S2. ROC Curve for association between maximum blood pressure and postnatal admission to neonatal ICU unit. AUC = 0.627 [0.538; 0.715], *p* < 0.009.
