# Supplementary material for: Perinatal Outcome and Its Association with Blood Pressure Levels in Women with Preeclampsia
Source: J Clin Med. 2022 Oct 27;11(21):6334. doi: 10.3390/jcm11216334 (PMC9654839; doi:10.3390/jcm11216334)
Supplement: Supplementary file 1 [file jcm-11-06334-s001.zip › jcm-1912461-supplementary.pdf]

Supplementary Table S1 Medication used for induction of labour. Shown is the mode of administration and the dosages used. Daily dosage was determined by the contraction pattern and the progress in labour.

Medication	Mode of Administration	Dosage
Misoprostol	oral	Starting dose: 25 µg<br>Continuing with 25 µg or 50 µg with a time interval of at least 4 hours
Dinoprostone	vaginal	Starting dose: 1 mg<br>Continuing with 1 mg or 2 mg with a time interval of at least 6 hours
Oxytocin	continuous infusion (intravenous)	Starting dose: 2 x 10^{-3} IU/min, stepwise increasing by 2 x 10^{-3} IU/min with a time interval of at least 15 min

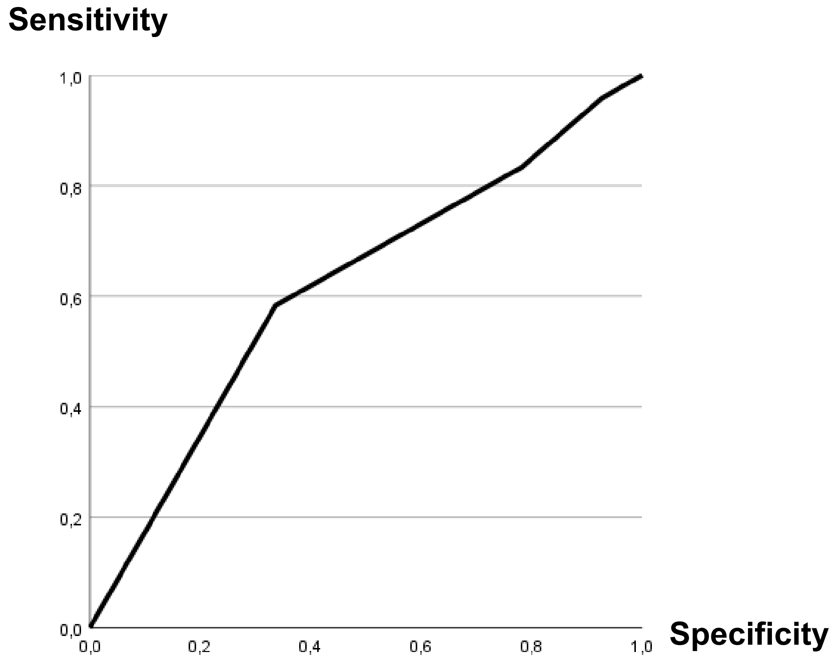

Supplementary Figure S1 ROC Curve for association between maximum blood pressure and gestational age at delivery. AUC = 0.615 [0.518 ; 0.713], p < 0.022

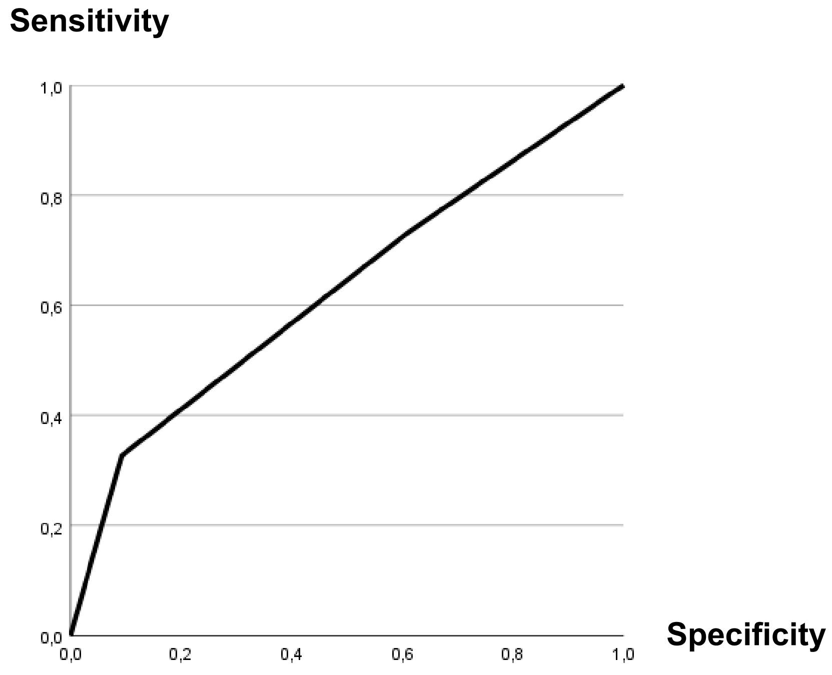

Supplementary Figure S2 ROC Curve for association between maximum blood pressure and postnatal admission to neonatal ICU unit. AUC = 0.627 [0.538 ; 0.715], p < 0.009
